# Neural Correlates of Verb Fluency Performance in Cognitively Healthy Older Adults and Individuals With Dementia: A Pilot fMRI Study

**DOI:** 10.3389/fnagi.2020.00073

**Published:** 2020-03-20

**Authors:** Eun Jin Paek, Laura L. Murray, Sharlene D. Newman

**Affiliations:** ^1^Department of Audiology and Speech Pathology, The University of Tennessee Health Science Center, Knoxville, TN, United States; ^2^School of Communication Sciences and Disorders, Western University London, London, ON, Canada; ^3^Department of Psychological and Brain Sciences, Indiana University, Bloomington, IN, United States

**Keywords:** dementia, neurodegenerative disease, verb fluency, action fluency, fMRI, neural correlates, hippocampus

## Abstract

Currently there are ~6 million Americans who are affected by dementia. Verbal fluency tasks have been commonly and frequently utilized to document the disease progression in many forms of dementia. Verb fluency has been found to display substantial potential to detect and monitor the cognitive declines of individuals with dementia who have fronto-striatal involvement. The neural substrates underlying verb fluency task performance, however, have remained unclear so far, especially in individuals with dementia. Therefore, in the current study, brain activation patterns of seven individuals with dementia and nine healthy older adults were investigated using functional MRI. The participants performed in the scanner an overt, subject-paced verb fluency task, representative of fluency tasks used in clinical settings. The brain activation patterns during the verb fluency task were compared between the two groups, and a correlational analysis was conducted to determine the neural correlates of verb fluency performance. The results suggest that compared to healthy older adults, individuals with dementia demonstrated poorer verb fluency performance and showed higher activation in specific neural regions, such as the bilateral frontal lobe. In addition, the correlational analysis revealed that poorer verb fluency performance lead to increased activation in certain cortical and subcortical areas, including left hippocampus and right supramarginal gyrus. The current findings are consistent with previous neurophysiological findings related to semantic (noun) fluency performance in older adults and individuals with dementia and add to the empirical evidence that supports the role of the frontal lobe and hippocampus in verb retrieval and search. Declines in verb fluency performance cannot only be used as a cognitive marker, but also represent neuropathological changes due to the neurodegenerative disease.

## Introduction

There are ~6 million Americans who live with dementia, with a substantial portion of people underdiagnosed and underreported (Alzheimer's Association, 2019). As there is yet no cure for many dementing diseases, early diagnosis, and prevention of disease progression is currently known to be the best management possible. Among many neuropsychological measures developed and validated to date for neurodegenerative diseases, verbal fluency tasks are one of the most commonly used and sensitive measures to detect and predict cognitive declines, given the high demands of these tasks on many cognitive processes such as semantic memory, strategic search, inhibition, and working memory, that are susceptible to the effects of dementing diseases (Henry et al., 2004; Belleville et al., 2017).

Numerous previous investigations have examined and supported the clinical utility and application of semantic (category) fluency, phonemic (letter) fluency, and action (verb) fluency tasks in the dementia population (e.g., Henry et al., 2004; Woods et al., 2005). All of these tasks require continued, effortful retrieval of distinct lexical items under certain restrictions (e.g., words that belong to a certain category or begin with a specific letter) in a given time period, with subtle differences among the different fluency tasks in terms of what cognitive components support their performance (see Henry et al., 2004 for a review). For instance, verb fluency (originally referred to as action fluency; see Piatt et al., 1999 for validity; Woods et al., 2005 for normative data) appears sensitive to cognitive deficits stemming from frontal-striatal impairments (Kochhann et al., 2018). Indeed, individuals with mild cognitive impairment (MCI) or subjective cognitive decline (SCD) have been found to show impaired verb fluency performance (Östberg et al., 2005, 2007; Forlenza et al., 2012; Alegret et al., 2018; Macoir et al., 2019) as have individuals with Alzheimer's disease (AD; Östberg et al., 2007; McDowd et al., 2011; Beber et al., 2015; Dubois et al., 2016; Alegret et al., 2018) and individuals with Parkinson's disease (PD) dementia (Piatt et al., 1999). Verb fluency tasks have also been used to predict dementia severity (e.g., Lai and Lin, 2013) and aid in the differential diagnosis of frontotemporal lobar degeneration (FTLD) vs. AD (Davis et al., 2010), AD vs. dementia with Lewy bodies (DLB; Delbeuck et al., 2013), and PD vs. PD with dementia (Piatt et al., 1999).

Brain imaging is commonly utilized as a biomarker for the detection of dementing, neurodegenerative diseases as well as to monitor disease progression, and biomarkers using functional MRI have been regarded useful in the diagnosis and intervention of dementia with substantial implications in delineating neuropathological changes (Gold and Budson, 2008; McKhann et al., 2011; Li et al., 2015; Bayram et al., 2018). Currently, however, there has been limited examination of how brains with neurodegenerative disease operate in relation to language and cognitive deficits (Szatloczki et al., 2015; Li et al., 2017; Kochhann et al., 2018). In particular, nominal research has focused on the neurophysiological correlates of impaired verbal fluency performance in the dementia population, despite the sensitivity, and popularity of verbal fluency tasks. In addition, there remains a significant gap in the extant literature with respect to verb fluency tasks, given that previous studies have been only focusing on measuring the neural responses with respect to semantic and phonemic fluency tasks in individuals with dementia.

To date, functional near-infrared spectroscopy (fNIRS) research related to dementia and verbal fluency has indicated that there are decreased hemodynamic responses in the inferior frontotemporal regions in the MCI population (Katzorke et al., 2018) and in the prefrontal and parietal areas in individuals with AD (Arai et al., 2006; Metzger et al., 2016) compared to healthy older adults, revealing the effects of neuropathological changes on brain activation patterns during verbal fluency tasks in these populations. The depth resolution of fNIRS, however, has limited the researchers to investigating relatively lateral brain regions that are more adjacent to the skull (Obrig, 2014); there has been little consideration of deeper brain structures (e.g., subcortical, medial, and insular areas as well as even some cortices), even though fluency tasks have been shown to engage these areas (Beber and Chaves, 2014; Li et al., 2017). In addition, the neural substrates supporting verb fluency performance in dementia remain elusive in that verb processing and noun processing are known to recruit not only shared, but also distinct brain areas (Davis et al., 2010; Beber and Chaves, 2014; Alyahya et al., 2018), possibly given the involvement of motor planning and sensorimotor systems related to action word processing (Bak, 2013; Horoufchin et al., 2018). Specifically, verb naming relies more on frontal-subcortical (frontal-striatal) circuits than noun naming.

Moreover, it is well-established that verb, semantic, and phonemic fluency tasks draw on different cognitive entities and anatomical neuronal substrates (Henry et al., 2004; Östberg et al., 2007; Stokholm et al., 2013; Clark et al., 2014; Faroqi-Shah and Milman, 2018; Gordon et al., 2018). For instance, verb fluency is known to engage syntactic processing as many verbs necessitate activation of argument structures related to thematic roles (Pekkala, 2012). That is, retrieval or activation of a verb (e.g., “give”) will likely activate the semantic representations of the agent, theme, and/or recipient associated with the target verb (agent—verb—theme—recipient; e.g., “Ethan gave water to Lilly”). Thus, the functional neural networks underlying verb fluency performance may be quite different from those underlying the other fluency tasks.

To date, nevertheless, there has been little fMRI research investigating the neural mechanisms supporting verb fluency task performance (Kochhann et al., 2018), and the literature is even more scarce regarding the population with dementia. The current literature for verb fluency in healthy populations or people with epilepsy indicates that the generation of verbs involves frontal-striatal areas including basal ganglia, dorsolateral prefrontal cortex, and left inferior frontal gyrus (Sanjuán et al., 2010; Benjamin, 2014). These investigations, however, have utilized experimental paradigms that either had the participants' verbal responses externally paced, or required participants to generate only one verb after a preceding stimulus (i.e., verb-verb or noun-verb generation tasks) and thus do not accurately reflect or represent the cognitive substrates underlying the verbal fluency task, which is one of the most commonly utilized paradigms due to its sensitivity to cognitive declines in neurodegenerative diseases as aforementioned.

There has also been limited investigation of the relationships between scores on the verb fluency task and neuroimaging parameters affected by neuropathological changes, such as degree of brain atrophy or hypoperfusion. A structural MRI study involving individuals with MCI or AD found that verb fluency performance correlated with brain volume in both hemispheres, including the LH dorsal frontal and temporal areas, and RH temporal, parietal, and occipital areas (Clark et al., 2014). A single-photon emission computed tomography study by Östberg et al. (2007) also reported that verb fluency scores were associated with bi-hemispherically represented neuronal substrates; more specifically, temporal lobe hypoperfusion (including temporal pole and medial temporal lobe) was associated with decreased verb fluency skills. These studies, however, sought to determine the relationship between the scores during a behavioral testing and neural structure or function, not directly accounting for the neural substrates (brain activity) underlying verb fluency performance. As functional neuroimaging investigations regarding those with neurodegenerative diseases are scant, questions remain regarding whether people with dementia exhibit decreased or increased activation in the same areas as healthy older adults do, or whether they show idiosyncratic, non-specific loci of activation as a sign of inefficiency or reduced specificity of brain function (Morcom and Henson, 2018). Delineating how damaged and healthy brains function during verb fluency will further enhance our understanding of the neural substrates for language and cognition in diseased populations and possibly identify distinct neural activation mechanisms that may be used as biomarkers for dementia.

Therefore, in the current pilot study, we tested individuals with dementia and cognitively healthy older adults (without a diagnosis of dementia) not only to compare their brain activation patterns, but also to determine brain regions closely associated with verb fluency performance. It was hypothesized that individuals with dementia will show significant upregulation of frontal lobe areas given the increased burden of search strategies dealing with degraded semantic information and access (Melrose et al., 2009; Reilly et al., 2011; Beber et al., 2015; Methqal et al., 2019); this hypothesis is also consistent with prior work documenting that impairments in verb fluency are associated with frontal lobe lesions (Piatt et al., 1999; Davis et al., 2010; Beber and Chaves, 2014). Additionally, it was hypothesized that there will be subcortical and deep brain regions exhibiting significant correlations with verb fluency performance. Specifically, we hypothesized that those regions affected early by the effects of dementia such as the hippocampus will be found to correlate with verb fluency performance because the hippocampus is known to contribute to the performance of other verbal fluency tasks (Glikmann-Johnston et al., 2015), and many types of dementias show hippocampal atrophy and dysfunction early in the disease process including FTLD, AD, and vascular dementia (Ostojic et al., 2015; Bede et al., 2018; Belkhelfa et al., 2018; Lladó et al., 2018).

## Materials and Methods

### Participants

A total of 16 older adults participated in the current fMRI study. Participants were recruited from the nearby communities, including dementia support groups, senior centers, and nursing facilities. This study is a part of a larger, longitudinal study project; described here is the project protocol related to the verb fluency task only, with other parts described elsewhere (Paek et al., 2019). The current study was reviewed and approved by Institutional Review Boards of Indiana University Bloomington and the University of Tennessee, Knoxville.

The inclusionary criteria for all participants included that each participant must be: (a) right-handed, (b) a native speaker of English, and (c) be able to participate in our longitudinal study over a 2-month span. Participants with dementia were given the diagnosis of dementia by their neurologists prior to their participation in the study. Individuals with any type of cortical dementia (including Alzheimer's dementia, vascular dementia, or FTLD, which causes primary progressive aphasia) of mild to moderate severity were included; prior research has shown these various dementia profiles are associated with decreased verbal fluency performance (Davis et al., 2010). In the current study, dementia severity was determined using the *Mini Mental Status Exam-2* (MMSE-2; Folstein et al., 2010): mild dementia if the obtained score (maximum 30) was equal to or higher than 21, and moderate dementia if the obtained score was equal to or higher than 11 (Perneczky et al., 2006).

Control participants had to demonstrate MMSE-2 scores higher than the cut-off score to participate in the study. The cut-off score for control participants was retrieved from the population-based norms of Crum et al. (1993), and the age- and education-level adjusted norms were used for each participant (e.g., 25 for individuals 80–85 years of age with 9–12 years of education).

The exclusionary criteria included that a participant should not have: (a) a history of epilepsy, psychiatric or developmental disorders, or other neurological disorders (e.g., epilepsy) than dementia, (b) claustrophobia, (c) any metal implants that have not been MR-safety approved by a doctor, (d) severe hearing or vision deficits that precluded completing tests and tasks, or (e) a significant level of depressive symptoms (i.e., Geriatric Depression Scale ≥ 10; Yesavage et al., 1983). Vision was screened through a picture-matching task, in which participants were required to match correctly a stimulus line drawing picture with an identical picture mixed with one foil picture. For the hearing-screening test, the Speech Discrimination Screening subtest from *Arizona Battery for Communication Disorders of Dementia* (ABCD; Bayles and Tomoeda, 1993) was given to all participants. In this task, participants were required to answer if the two words that they just heard were the same or not. For the vision and hearing screening tests, at least 70% accuracy was required to participate in the current study. Participants also completed the Geriatric Depression Scale (GDS) to screen for severe depression (Yesavage et al., 1983).

Seven individuals with dementia and nine age- and education-matched healthy adults participated and completed all procedures in this study (see Table 1). Five participants had an AD diagnosis, one a vascular dementia diagnosis, and one a FTLD diagnosis (i.e., non-fluent variant primary progressive aphasia). Between the dementia group and the control group, there was no statistically significant difference in terms of age, *t*(14) = −0.74, *p* =.47, level of education *t*(14) = −0.15, *p* = 0.88, or sex χ^2^ (1, *N* = 16) = 0.04, *p* = 0.84, whereas the MMSE-2 scores were significantly higher for the control group compared to the dementia group *t*(14) = 0.36, *p* = 0.00.

**Table 1:** Participants' demographic information.

**Participant**	**Diagnosis**	**Age**	**Education**	**Sex**	**MMSE-2 raw score**	**GDS raw score**
C1	None	64	12	M	28	0
C2	None	64	14	F	30	0
C3	None	83	17	M	29	0
C4	None	66	16	M	28	0
C5	None	67	18	F	30	0
C6	None	67	18	F	30	0
C7	None	68	16	F	28	1
C8	None	68	18	F	30	0
C9	None	66	16	F	27	0
Control group mean (SD)		70.57 (7.55)	16.11 (2.69)	3 male, 6 female	28.89 (1.09)	0.11 (0.33)
D1	AD	77	18	F	29	2
D2	nfvPPA	67	16	F	22	3
D3	AD	65	14	F	24	6
D4	AD	69	16	F	28	0
D5	VaD	74	18	M	23	2
D6	AD	82	20	M	23	3
D7	AD	60	12	F	16	4
Dementia group mean (SD)		68.11 (5.78)	16.29 (2.03)	2 male, 5 female	23.57 (4.28)	2.85 (1.86)

*AD, Alzheimer's disease; GDS, Geriatric Depression Scale; MMSE-2, Mini Mental Statue Exam-2; nfvPPA, non-fluent variant primary progressive aphasia; SD, standard deviation; VaD, vascular dementia*.

## fMRI Stimuli and Paradigms

The entire neuroimaging session was comprised of five functional runs and one anatomical run, but only the runs related to the anatomical scans and the verb fluency task are described in this study (see Paek et al., 2019 for details about other parts of the session). It is well-established that verbal fluency tasks are sensitive to dementia (e.g., Östberg et al., 2005), and previous studies have shown that fMRI of word production is feasible and can yield reliable results (e.g., Birn et al., 2004; Marsolais et al., 2015). All participants previously experienced an MRI scan and performed in-scanner naming tasks as part of the larger project; thus, they were familiar with the task and situation. All participants practiced the naming tasks before going into the scanner, and additionally they were instructed immediately before each functional MRI run that they would be performing a verb fluency task using the intercom in the scanner. Such practice and instructions were utilized to ensure that participants could perform the task without disorientation.

During functional runs, participants were shown a fixation cross on the screen for 30 s to establish their baseline brain activation at the beginning of the run. Then written instructions for the verb fluency task were given for 10 s, asking the participants to name as many words as possible that people do in a 30-s period (i.e., “Tell me as many words as possible that people “do” in one word. For example, eat, smell, and so on. Ready?”). Participants were also asked not to use the same word with different endings such as “eat,” “eating,” or “eaten;” no participant in our study generated this type of error. This 30-s block of verb fluency (Destrieux et al., 2012) was repeated three times in one run, and each trial alternated with a 10-s resting period. For the second and third trials, participants were also asked not to repeat the verbs that they already said in the previous trial so that for each trial they could generate a unique set of verbs. That is, in the written instructions, it was added “do not repeat the words that you already said before.” An unpaced, overt generation paradigm was utilized in the present study (Birn et al., 2010) so that the task was similar to what is clinically utilized for people with dementia, and also to avoid the confounding extra-linguistic factors that paced and covert paradigms may introduce, such as divided attention, response conflict, and inhibition (Basho et al., 2007).

## Data Acquisition and Analysis

All fMRI scanning was conducted on a Siemens 3T TIM Trio scanner with a 32-channel radio frequency head coil in the Imaging Research Facility at Indiana University. In each session, functional images were obtained in 38 oblique axial slices with 3 mm thickness (TR = 2,500 ms and TE = 27 ms, flip angle = 70, matrix size = 64 × 64, FOV = 240 mm) using a gradient echo planar imaging (EPI) sequence in an interleaved manner. A 3D structural image was also taken using a sagittal T1-weighted sequence with an MPRAGE sequence (TR = 1,800 ms, TE = 2. 7 ms, flip angle = 9, matrix size = 256 × 256, FOV = 256 mm, 192 sagittal slices, slice thickness = 1 mm). All functional images were preprocessed using SPM12 (Wellcome Department of Imaging Neuroscience), including steps of slice timing correction, motion correction (realignment), spatial normalization, coregistration using the Montreal Neurological Institute (MNI) template, and smoothing (FWHM = 6 mm). The processed images were entered into the statistical analysis based on the general linear model and the Gaussian random field theory, which are implemented in the SPM package. For the verb fluency paradigm, response onset times for each response from the three trials and the baseline period were entered as regressor. The motion parameters were entered in the design matrix as variables of no interest to reduce the impact of head position. Additionally, given that our experiment requires participants' overt verbal responses, we analyzed the amount of head motion to determine if there were significant differences between the groups. First, the motion parameters for the x, y, z axes were extracted and converted into absolute numbers and then averaged for each participant. Then *t*-tests were conducted for each of the parameters. The participants with dementia had slightly larger movements than the control participants, but these differences were not significant statistically (see Supplementary Table 1).

Participants' overt responses were recorded in the scanner using an in-house MR-compatible microphone system while they were performing the verb fluency task. The responses recorded during the verb fluency task were transcribed and recorded in terms of the number of correct responses per trial. The number of correct responses per trial were compared between groups using independent samples *t*-tests. Also, a repeated measures ANOVA was performed to compare the groups' verb fluency performances over the three trials, followed by *post hoc* pairwise comparisons using Bonferroni tests. Statistical analyses of these behavioral data were conducted using SPSS.

To detect brain areas activated by the verb fluency task, multiple regression, and general linear tests were performed on each participant's data. Verb fluency responses across all three trials were collapsed for each participant, generating [verb fluency > rest] for every participant. At the group level, one sample *t*-tests were computed to examine the neural correlates of verb fluency performance in each group, and then two sample *t*-tests were performed to determine differences in brain activation between the control (*n* = 9) and dementia groups (*n* = 7) relative to verb fluency performance using the age and education level as covariates. Additionally, to delineate the neural regions associated with the level of performance on the verb fluency task, linear regression analysis was used with the total number of correct verb produced as a covariate of interest across all 16 participants using the [verb fluency > rest] contrast images. As dementia progresses, decreased performance on verbal fluency tasks is observed, and verbal fluency deficits are known pathological markers of cognitive declines due to dementing diseases, even though the deficits are also observed in subclinical populations (Clark et al., 2009; see Belleville et al., 2017; Nikolai et al., 2018). Furthermore, structural and functional neuroimaging studies related to verbal fluency tasks in individuals with MCI or dementia have suggested that cognitively healthy older adults and individuals with dementia exhibit similar brain activation patterns or structural brain changes, differing only in terms of level of activation (i.e., decreased activation in AD; Arai et al., 2006; Metzger et al., 2016) or severity of structural or perfusion changes (Östberg et al., 2007; Metzger et al., 2016; Rodríguez-Aranda et al., 2016). Thus, we utilized a multiple regression approach for the fMRI BOLD responses on the continuum from cognitively healthy older adults to individuals with moderately severe dementia to capture the neural substrates underlying impaired verb fluency performance. All SPM(*t*) maps were thresholded and then overlaid onto a template brain.

## Results

### Behavioral Results

**Figure 1:**
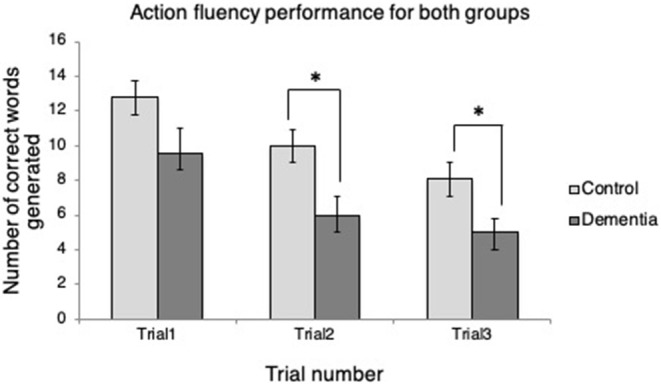
Verb fluency performance across trials for each group. *p < 0.05.

Independent samples *t*-tests revealed that the two groups differed in the number of words generated in two of three trials (Figure 1). At Trial 1, the control group produced more verbs (*M* = 12.78, *SD* = 2.99) than the dementia group (*M* = 9.57, *SD* = 3.69), but the difference between the groups was not statistically significant, *t*_(14)_ = 1.92, *p* = 0.075. At Trial 2, the control group produced significantly more verbs (*M* = 10.00, *SD* = 2.78) than the dementia group (*M* = 6.00, *SD* = 2.77); *t*_(14)_ = 2.86, *p* = 0.013. Similarly, at Trial 3, the control group produced significantly more verbs (*M* = 8.11, *SD* = 2.89) than the dementia group (*M* = 5.00, *SD* = 2.00); *t*_(14)_ = 2.24, *p* = 0.030. In addition, it was examined whether the two groups' verb fluency performance differed over time in terms of the number of correct words generated. Mauchly's Test of Sphericity indicated that the null hypothesis could be retained (*p* = 0.148). Repeated measures ANOVA revealed that there were effects of time, *F*_(2, 13)_ = 16.05, *p* < 0.001. Bonferroni correction for adjustment for multiple comparisons indicated that the participants produced fewer and fewer words over the repeated trials: More verbs were generated in Trial 1 than Trial 2 (*p* < 0.001), in Trial 2 than Trial 3 (*p* = 0.028), in Trial 1 than Trial 3 (*p* < 0.001). There was, however, no time by group interaction, *F*_(2, 13)_ = 0.518, *p* = 0.607.

## Neuroimaging Results

### Whole Brain Voxel-Wise Analysis Results for Each Group

**Figure 2:**
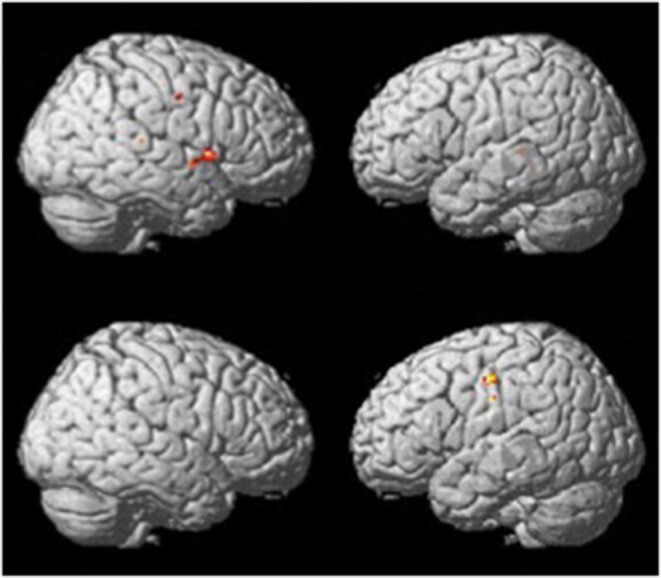
Whole brain activation patterns for verb fluency for the control group (top) and dementia group (Bottom).

The verb fluency paradigm (thresholded at *p* <.001, uncorrected; see Figure 2) yielded activation in the RH precentral gyrus in both groups. Compared to the resting period, the control group during verb fluency demonstrated activation in: right hemisphere (RH) inferior frontal gyrus (IFG), RH Rolandic operculum, RH superior temporal gyrus (STG), RH temporal pole, RH cerebellum, left hemisphere (LH) middle temporal gyrus (MTG), and LH insula. Participants with dementia showed distinctive activation only in the bilateral postcentral gyrus during verb fluency compared to their resting period.

### Group Differences

To determine if there were any group differences in activations during the verb fluency task, two-sample *t*-tests were administered with the age and education level as covariates (at *p* = 0.005*, k* >= 10). This group level comparison revealed that the two groups activated distinctive brain areas for the verb fluency task (Table 2). Specifically, compared to the control participants, individuals with dementia activated bilateral superior frontal gyrus (SFG), pars triangularis of IFG, LH supplementary motor area, RH middle frontal gyrus (MFG), and RH pars opercularis of IFG. Compared to the dementia group, the control group presented with activation in the bilateral precentral gyrus, LH insula, LH Rolandic operculum, and RH calcarine and precuneus.

**Table 2:** Group differences between individuals with dementia and cognitively healthy older adults for verb fluency performance and correlation results.

**Cluster size**	**Overlap of cluster with anatomical region(s)**	**peak MNI coordinate**			**T_**max**_**
		**x**	**y**	**z**	
Control Group > Dementia Group					
52	Calcarine, precuneus_R	32	−50	8	4.5666
19	Rolandic_Oper_L, Insula_L	−40	10	14	3.9834
13	Precentral_R	40	−26	56	3.6107
12	Precentral_L	12	−26	60	4.1669
11	Rolandic_Oper_L, Insula_L	−42	−2	10	3.7511
Dementia Group > Control Group					
31	Frontal_Mid_R, Frontal_Inf_Oper_R	36	14	40	4.2968
24	Frontal_Sup_L	−12	20	56	5.8877
23	Frontal_inf_Tri_R	52	24	20	6.3753
19	Frontal_Sup_L, Supp_Motor_Area_L	−18	2	62	4.4732
14	Frontal_inf_Tri_L	−42	30	28	3.4195
11	Frontal_Sup_R	22	4	52	4.9149
Negative Correlation of Brain Activation Regions With Number of Words Generated					
81	Hippocampusl_L, Precuneus_L, Thalmus_L	−18	−40	6	4.0346
63	Supramarginal_R, Postcentral_R, Rolandic_Oper_R	62	−22	24	4.1040
58	Supramarginal_R, Rolandic_Oper_R	42	−34	24	5.5598

*The table provides the anatomical localization of each cluster and lists the overlap with cytoarchitectonically defined areas. References to cytoarchitectonic maps are based on AAL (Tzourio-Mazoyer et al., 2002). Tmax, T value at local maximum*.

### Linear Regression Results

**Figure 3:**
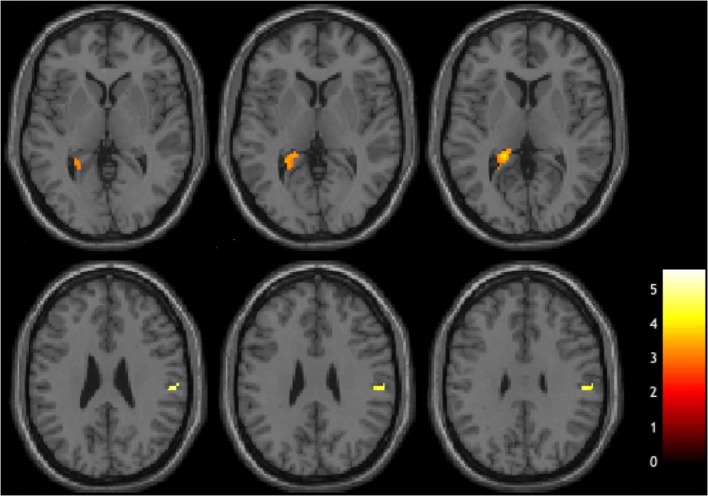
Brain regions showing negative correlations with verb fluency task.

**Figure 4:**
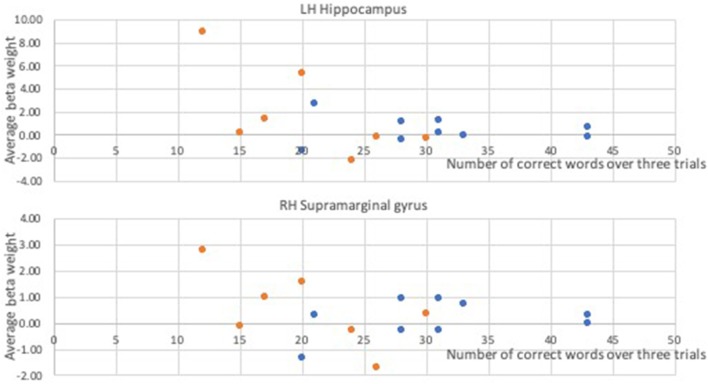
Correlational relationships between the BOLD response and the number of correct verb fluency responses. Orange dots, participants with dementia; blue dots, control participants.

In a whole-brain linear regression analysis at *p* < 0.005, *k* >= 50, a significant negative correlation between the BOLD responses and participants' verb fluency performances was observed in the LH hippocampus area. Activation magnitudes were higher for those participants with poorer verb fluency scores. Other regions showing a negative correlation included RH supramarginal gyrus as well as small areas within RH postcentral gyrus and Rolandic operculum (see Table 2 and Figures 3, 4). To test the generalizability of these results, a secondary analysis was undertaken while removing two individuals with AD randomly from the analysis. This approach was utilized because the original dementia sample was relatively skewed to the AD diagnosis or profile. That is, we aimed to examine whether reducing this skew of AD profile representation would influence the pattern of regression results; if removing these two individuals didn't change the results, we could have more confidence in the generalizability of our findings to a the broader array of dementia types associated with verb fluency deficits. Indeed, the results were similar, showing that the activation in the LH hippocampus areas and RH supramarginal gyrus areas was associated with poor verb fluency performance (see Supplementary Table 2).

## Discussion

The current pilot fMRI study examined the neural correlates of an overt verb fluency task in healthy aging adults and individuals with dementia. Individuals with dementia generally produced fewer correct responses during the repeated verb fluency tasks compared to the cognitively healthy older adults. There was no interaction of group and time, however, indicating that both groups demonstrated a comparable degree of decrement in their verb fluency responses as they progressed from the first through to the third and final trial. During the verb fluency task various brain areas were recruited in our participants, as verbal fluency tasks not only require executive functioning and search strategies to retrieve target words, but also includes morphophonological encoding, articulation, and self-monitoring (Birn et al., 2010; Pekkala, 2012). As predicted, compared to cognitively healthy older adults, increased activation was observed in the frontal regions in individuals with dementia during the verb fluency task. It was also revealed that increased activation of LH hippocampus and some additional cortical areas including RH supramarginal gyrus were associated with poorer verb fluency performance in both healthy aging and dementia. The implications of these findings are discussed below.

### Brain Activation Differences Between Cognitively Healthy Older Adults and Individuals With Dementia

During the verb fluency task, there were several brain regions that were more activated in the healthy group compared to the dementia group. These regions included bilateral precentral gyri, precuneus and calcarine areas, as well as LH insula. Increased activation in the precentral areas is possibly attributable to increased speech motor planning or articulatory movements given the higher number of responses generated by the healthy compared to the dementia group. Likewise, prior research in healthy adults has indicated that verbal fluency tasks yield significant activation in these regions (Paulesu et al., 1997; Pihlajamäki et al., 2000; Meinzer et al., 2009, 2012; Birn et al., 2010; Nagels et al., 2011; Marsolais et al., 2015; Li et al., 2017). As the verb fluency task requires participants to retrieve lexical items that are specifically pertinent to actions that people do, visual imagery of these verbs during search or retrieval may have led to the activation of precuneus and calcarine areas. Precuneus engages during a wide range of cognitive tasks, including visuo-spatial imagery and memory retrieval (Cavanna and Trimble, 2006), and similarly the calcarine cortex is known to contribute to visual imagery processes as well (Klein et al., 2000). Given that these areas were found to be utilized more in the cognitively healthy older adults (who have preserved verb fluency performance) than the individuals with dementia, this finding may suggest that verb fluency task performance within the control group was supported by visual imagery of the generated verbs more than the dementia group.

In contrast, in individuals with dementia there was increased activation in the bilateral SFG and IFG compared to the cognitively healthy older adults. Extensive activation in frontal lobe areas during verbal or orthographic fluency tasks has been commonly observed in many neuroimaging studies involving healthy adults (e.g., Pihlajamäki et al., 2000; Costafreda et al., 2006; Meinzer et al., 2009, 2012; Birn et al., 2010; Nagels et al., 2011; Marsolais et al., 2015). Also, bilateral frontal areas are reportedly a common ground brain network recruited during different types of verbal fluency tasks and word retrieval (e.g., fill-in-the-blank sentence completion) in healthy young adults (Li et al., 2017). The current findings in which individuals with dementia demonstrated upregulation of these frontal lobe areas may indicate (a) compensatory mechanisms utilized to generate as many words as possible in the face of decreased cognitive and linguistic resources, or (b) neural maladaptation or inefficiency, with frontal involvement being detrimental to verb fluency performance (Morcom and Henson, 2018; Methqal et al., 2019). Although we cannot distinguish between these two hypotheses in the current study, our findings suggest that despite higher bilateral frontal lobe activation among individuals with dementia, these participants still demonstrated poorer performance compared to cognitively healthy older adults, supporting the previous findings of Meinzer et al. (2009, 2012) and consistent with the CRUNCH (Compensation-Related Utilization of Neural Circuits Hypothesis; Reuter-Lorenz and Cappell, 2008) effects. The critical influence of cognitive demands and task difficulty on RH brain activity has been investigated by Meinzer et al. (2009, 2012) who found that increased RH inferior frontal activation was related to decreased semantic and phonemic fluency performances in young and older adults. These findings align with our results as our participants with dementia who produced a smaller number of correct verb fluency responses, more strongly activated their RH frontal lobe regions as well as LH frontal areas, possibly due to increased demands for cognitive control. That is, our findings may also suggest further utilization of executive functioning skills, specifically strategic search (Birn et al., 2010; Li et al., 2017), by individuals with dementia, to find and retrieve the target verbs relative to cognitively healthy older adults. People with dementia not only lose control of attentional and executive functioning skills as their dementing disease progresses, but also demonstrate significant semantic degradation and impaired access to semantic representations (Reilly et al., 2011; Stopford et al., 2012; Szatloczki et al., 2015; Belleville et al., 2017; Slegers et al., 2018). The up-regulation of frontal lobe areas may indicate additional burden to support attention and executive functions (i.e., search strategies) and organization of semantic clusters to keep up their verb fluency performance in the midst of a loss of semantic information.

### The Involvement of Subcortical Structures and Medial Temporal Lobe for Verb Fluency

The current study also scrutinized whether specific brain regions exhibit correlational activation with performance levels on the verb fluency task in aging and dementia populations. Subcortical networks demonstrated significant correlations with poor verb fluency performance, including the hippocampus, precuneus and thalamus in LH. The RH supramarginal gyrus areas also showed significant negative correlations with verb fluency performance. Verbal fluency tasks, in general, are highly multi-faceted in that they require many different cognitive processes to be engaged simultaneously; this demand on diverse cognitive abilities may explain why the sensitivity of this task to the presence of dementia is high (Belleville et al., 2017). The completion of verbal fluency tasks demands not only semantic memory and vocabulary, but also controlled, effortful search and retrieval of semantic memory, working memory, processing speed, and other executive function skills such as inhibition and self-monitoring (Woods et al., 2005; McDowd et al., 2011; Stolwyk et al., 2015). Thus, various brain areas responsible for these cognitive processes would need to be actively recruited when a person performs a verbal fluency task, and our current finding of a contribution from the hippocampus as a “non-language” structure (Glikmann-Johnston et al., 2015) is not unexpected. For example, medial temporal lobe (MTL) and adjacent areas have been reported to be integral to semantic networks, and activations in these regions have been found to be significantly associated with semantic fluency performance given their role in extracting information from semantic storage (Pihlajamäki et al., 2000; Glikmann-Johnston et al., 2015; Kochhann et al., 2018; Nikolai et al., 2018). In the current study, the upregulation of MTL and related regions in those who showed poorer verb fluency performance may have been due to the concurrent recruitment of brain areas responsible for language and cognitive abilities (Li et al., 2017), which are important for fluency.

Additionally, given that performance of semantic fluency tasks, but not phonemic fluency tasks, has been found to involve MTL activity (Glikmann-Johnston et al., 2015), the current neural finding of hippocampal involvement in verb fluency performance may support that the cognitive substrates of the verb fluency task are more similar to those of a semantic fluency vs. a phonemic fluency task. That is, both the cognitive and neural processes required for an verb fluency task resemble those for a semantic fluency task, more so than a phonemic fluency task. (Glikmann-Johnston et al., 2015) had healthy young adults complete semantic and phonemic fluency tasks and then performed functional connectivity analyses; these researchers reported that hippocampal activation was an integral part of the neural network supporting semantic fluency but not phonemic fluency task performance. That is, there was high connectivity of the hippocampus with the semantic neural networks when the healthy young adults were performing the semantic fluency task. Although verb fluency task is regarded as a measure of executive functioning skills and frontal lobe deficits, more so than semantic or phonemic fluency tasks (Woods et al., 2005), the semantic constraints that limit the participants to name only verbs during the task (e.g., perhaps approached by participants as a type of semantic category) may have made this task have a common neural involvement of the hippocampus like semantic fluency tasks. On the other hand, there is relatively less for verb fluency to share with the cognitive and neural substrates supporting phonemic fluency task performance, which has been found to be heavily dependent on the orthographic search for target stimuli (Birn et al., 2010).

On the other hand, the negative correlation between RH supramarginal gyrus and verb fluency performance was not specifically predicted prior to the experiment given this region's classic role in and support of phonological processing and articulatory rehearsal (Price, 2012). For example, Birn et al. (2010) reported that an automatic speech task (i.e., citing months of the year) yielded increased activity of RH supramarginal gyrus in healthy young adults compared to semantic and phonemic fluency activation patterns. The active involvement of RH supramarginal gyrus, however, has been found in other previous studies of verbal fluency or object naming in older adults and individuals with dementia (Wierenga et al., 2008; Marsolais et al., 2015). Furthermore, Meinzer et al. (2012) found that compared to cognitively healthy older adults, RH supramarginal gyrus showed negative activity in healthy young adults during an externally paced verbal fluency paradigm. Consistent with the current results, Meinzer et al. found that producing more correct responses was associated with more negative activity in RH supramarginal gyrus. Thus, the current results add to the evidence base that poorer verbal fluency performance is linked to more activation in the RH supramarginal area.

### Study Limitations

The results of the current pilot study should be interpreted in light of some limitations. First, the current experimental paradigm did not allow controlling the total number of responses for each of the verb fluency trials. Thus, there was a greater number of words analyzed in the fMRI data of the healthy compared to the dementia group. This is inevitable, however, when utilizing a pace-free fluency task. If word generation is paced, more cognitive processes, such as inhibition, actively engage to meet the needs of the pacing requirement (Basho et al., 2007; Marsolais et al., 2015). Also the standard verbal fluency task demands are not reflected with a paced verbal fluency task (Nagels et al., 2011). Thus, the current study utilized the conventional paradigm that is actually used in, and more representative of most clinical and research settings (Nagels et al., 2011; Marsolais et al., 2015). One advantage of utilizing this pace-free fluency task is that, unlike other previous studies related to action semantics in dementia (e.g., Bak, 2013), there is no need to control for possible confounding factors such as word frequency and age of acquisition. Additionally, the interpretation of the results considered the number of words produced and the possible impact of such differences as aforementioned in our discussion.

Another limitation relates to the heterogeneity in the dementia group, although the majority of participants had a diagnosis of AD; there were 5 participants with AD, 1 with vascular dementia, and another with FTLD. Advances in neuropathology research have led researchers to consider the substantial impact of various neuropathologies on the relationships between a certain type of dementia and its clinical symptoms. However, even though our sample likely had heterogeneous neuropathology, they all showed prominent language declines and verb fluency performance deficits; likewise, all of these dementia types are characterized by cognitive and linguistic deficits, which in turn contribute to communication problems and limitations in daily activities (Hickey and Bourgeois and Hickey, 2009; McKhann et al., 2011). Thus, the results of this pilot study contribute to understanding the relationship between decreased cognitive performance and brain activation patterns, namely, neural correlates. Along the same vein, given the small sample size, statistical power was relatively limited. To the best of our knowledge, however, this is the first fMRI study that used an overt, free-generation (unpaced) verb fluency task to examine the neural substrates underlying verb fluency performance in the dementia population. Beber and Chaves (2014) pointed out that the clinical findings of impaired verb fluency performance among individuals with various dementias corroborate the frontal circuitry deficits observed in many neurodegenerative diseases including FTLD, AD, MCI, and PD. The present findings hopefully provide the groundwork to further investigate this topic given that our dementia participants exhibited substantially increased frontal activation in both hemispheres compared to cognitively healthy older adults. Further investigation is warranted to delineate the effects of dementia type and a broader range of dementia severities on verb fluency performance and neural correlates.

## Conclusion

The complex task demands of verb fluency tasks require efficient use of many cognitive and linguistic skills, and individuals with dementia exhibit deficits in verb fluency performance early and prominently. The current study findings suggest that individuals with dementia demonstrate upregulation of bilateral frontal areas compared to cognitively healthy older adults when completed verb fluency tasks. Additionally, higher activation of MTL and other cortical areas was associated with poor verb fluency performance in both cognitively healthy older adults and those with dementia. Thus, declines in performance of verb fluency tasks can be used as not only a cognitive marker, but also a neural marker of frontal lobe deficits observed in neurodegenerative diseases.

## Data Availability Statement

The datasets generated for this study are available on request to the corresponding author.

## Ethics Statement

The studies involving human participants were reviewed and approved by Indiana University Institutional Review Board. The patients/participants provided their written informed consent to participate in this study.

## Author Contributions

EP, SN, and LM contributed to the conception, design, and data collection of the study and wrote sections of the manuscript. EP and SN performed the statistical analyses. EP wrote the first draft of the manuscript. All authors contributed to manuscript revisions, and read and approved the submitted version.

### Conflict of Interest

The authors declare that the research was conducted in the absence of any commercial or financial relationships that could be construed as a potential conflict of interest.
