# Supplementary material for: Neural Correlates of Verb Fluency Performance in Cognitively Healthy Older Adults and Individuals With Dementia: A Pilot fMRI Study
Source: Front Aging Neurosci. 2020 Mar 20;12:73. doi: 10.3389/fnagi.2020.00073 (PMC7100367; doi:10.3389/fnagi.2020.00073)
Supplement: Supplementary file 1 [file Table_1.docx]

Supplementary Table 1.

Comparison of head motion parameters between the dementia and control groups

|  | |  |  |  |  |  |  |
| --- | --- | --- | --- | --- | --- | --- | --- |
|  | group | | Mean | Standard deviation | *df* | *t* | *p* |
| x | control | | 0.11 | 0.08 | 14.00 | -1.18 | 0.26 |
|  | dementia | | 0.15 | 0.08 |  |  |  |
| y | control | | 0.10 | 0.08 | 14.00 | 1.20 | 0.25 |
|  | dementia | | 0.15 | 0.08 |  |  |  |
| z | control | | 0.27 | 0.20 | 14.00 | -1.08 | 0.30 |
|  | dementia | | 0.42 | 0.32 |  |  |  |

Supplementary Table 2.

Correlation results for verb fluency performance and brain activation regions

| Cluster size | Overlap of cluster with anatomical region(s) | peak MNI coordinate | | | T_max_ |
| --- | --- | --- | --- | --- | --- |
|  |  | x | y | z |  |
| Negative correlation of brain activation regions with number of correct words generated (*p* < .005, k >= 10) | | | | | |
| 163 | Cingulate gyrus_R | 12 | 4 | 26 | 4.802 |
| 97 | Hippocampus_L, Precuneus_L | -18 | -40 | 6 | 3.523 |
| 76 | SupraMarginal_R | 42 | -34 | 24 | 5.982 |
| 61 | Insula_L, Rolandic_Oper_L | -42 | -8 | 8 | 4.296 |
| 32 | Insula_L | -38 | 6 | 6 | 4.668 |
| 24 | Insula_R | 46 | 12 | -4 | 3.859 |
| 19 | Precentral_R | 58 | 6 | 30 | 3.628 |
| 17 | Angular_L | -50 | -58 | 26 | 5.279 |
| 16 | Rolandic_Oper_R | 46 | -12 | 16 | 4.484 |
| 15 | SupraMarginal_R | 62 | -22 | 24 | 4.120 |

*Note*. The table provides the anatomical localization of each cluster and lists the overlap with cytoarchitectonically defined areas. References to cytoarchitectonic maps are based on AAL (Tzourio-Mazoyer et al., 2002). Tmax = T value at local maximum.
